# Ticagrelor Versus Prasugrel in Acute Coronary Syndrome: Real-World Treatment and Safety

**DOI:** 10.3390/medicines12020013

**Published:** 2025-05-14

**Authors:** Fadel Bahouth, Boris Chutko, Haitham Sholy, Sabreen Hassanain, Gassan Zaid, Evgeny Radzishevsky, Ibrahem Fahmwai, Mahmod Hamoud, Nemer Samnia, Johad Khoury, Idit Dobrecky-Mery

**Affiliations:** 1 Department of Cardiology, Bnai-Zion Medical Center, Haifa 31096, Israel; 2 Pulmonology Division, Carmel Medical Center, Haifa 3436212, Israel

**Keywords:** acute coronary syndrome, ticagrelor, prasugrel, dual anti platelet drugs

## Abstract

**Introduction**: A direct head-to-head comparison between potent P2Y12 inhibitors: prasugrel versus ticagrelor is still lacking. **Purpose**: In this single-center study, we sought to address the efficacy and safety of these two third-generation antiplatelet drugs, after about a decade of practical use. **Methods**: A retrospective observational study included all patients who were admitted with acute coronary syndrome between January 2010 and December 2019 and were discharged with aspirin and either prasugrel or ticagrelor after percutaneous coronary intervention. Patients were divided into two groups based on the dual antiplatelet drugs prescribed. Primary endpoint: A composite endpoint of cardiovascular death, recurrent coronary syndrome, or ischemic stroke at one year. Secondary endpoint: Significant bleeding according to the BARC classification (types 3, 4, or 5). **Results**: During this period, 746 patients met the inclusion criteria. The primary endpoint was reached in 70 patients (9.4%): 24 patients (8.0%) in the group treated with ticagrelor and 46 patients (10.3%) in the group treated with prasugrel (*p*-value = 0.303). In terms of safety events, significant bleeding was not statistically different between the ticagrelor and prasugrel groups: 13 (2.9%) vs. 9 (3%), respectively (*p*-value = 0.9). More patients discontinued their treatment before the end of the year among those treated with ticagrelor compared to those treated with prasugrel (16.7% vs. 9.6%, *p*-value = 0.003). **Conclusions**: There was no significant difference in the occurrence of recurrent cardiac events, stroke, or cardiovascular death, nor significant bleeding among ACS patients treated either with prasugrel or ticagrelor.

## 1. Introduction

Cardiovascular disease (CVD) is the most common cause of mortality and morbidity, contributing to approximately 640,000 deaths per year in the United States [1]. It is also the most common cause of mortality worldwide, with a substantial portion of this burden borne by low- and middle- income countries. Acute coronary syndrome (ACS) is often the first clinical manifestation of CVD.

Ischemic heart disease is the most common cause of CVD death, accounting for 38% of all CVD deaths in females and for 44% in males. Based on the most recent ESC guidelines, ACS encompasses a spectrum of conditions that include patients presenting with recent various chest symptoms, mainly chest pain or dyspnea and/or signs of acute heart failure, with or without changes on a 12-lead electrocardiogram (ECG) and with or without acute elevations in cardiac troponin (cTn) concentrations. Patients presenting with suspected ACS may eventually receive a diagnosis of acute myocardial infarction (AMI) or unstable angina (UA). The diagnosis of myocardial infarction (MI) is associated with evidence of myocardial injury and necrosis that results in serum cardiac troponin elevation and is made based on the fourth universal definition of MI. Unstable angina pectoris is defined as a myocardial ischemia that occurs at rest or minimal exercise in the absence of myocardial injury [1,2].

Dual antiplatelet therapy (consisting of an adenosine diphosphate receptor antagonist and aspirin–DAPT) is the standard treatment for patients with acute coronary syndromes (ACSs). For more than a decade, invasive coronary angiography maintains its central role in the management of patients with myocardial infarction [2,3,4].

The combination of two drugs from two different antiplatelet drug groups—aspirin (acetylsalicylic acid), which irreversibly inactivates cyclooxygenase (COX) activity, [5] and P2Y12 inhibitors (clopidogrel, prasugrel, or ticagrelor), which prevent the activation of ADP and thus inhibit platelet aggregation—inhibits platelet aggregation in a more potent way than single-drug therapy [6].

For years, patients with ACS were recommended aspirin use and clopidogrel for one year, based on the Clopidogrel in Unstable Angina to Prevent Recurrent Events (CURE) study, until the TRITON-TIMI 38 and PLATO studies demonstrated the superiority of a prasugrel- and ticagrelor-based regimen, respectively, over a clopidogrel-based one [5,6,7,8,9]. Prasugrel and ticagrelor provide greater, more rapid, and more consistent platelet inhibition than their predecessor clopidogrel [7,8,9].

The Trial to Assess Improvement in Therapeutic Outcomes by Optimizing Platelet Inhibition with Prasugrel–Thrombolysis in Myocardial Infarction (TRITON-TIMI 38) included P2Y12 inhibitor-naive ACS patients who were scheduled for PCI and received the drugs (clopidogrel or prasugrel) during or after the PCI. The composite primary endpoint (cardiovascular death, non-fatal MI, or stroke) occurred in 9.3% of prasugrel-treated patients vs. 11.2% of clopidogrel-treated patients (*p*-value 0.002), mostly driven by a significant risk reduction for MI, including in-stent thrombosis. There was no difference in the rates of either non-fatal stroke or cardiovascular death.

Prasugrel was associated with a significant increase in the rate of bleeding, including major and life-threatening bleeding, compared to clopidogrel [6,8,9,10,11,12].

In the PLATelet inhibition and patient Outcomes (PLATO) trial, 18624 patients with moderate- to high-risk NSTE-ACS (planned for either conservative or invasive management) or ST-segment elevation myocardial infarction (STEMI) were randomized to receive either clopidogrel or ticagrelor. In the NSTE-ACS subgroup (n = 11080), the primary composite efficacy endpoint (death from CV causes, MI, or stroke) was significantly reduced with ticagrelor compared to clopidogrel (10.0% vs. 12.3%; HR 0.83 (95% CI 0.74, 0.93), *p* = 0.0013), with similar reductions for CV death (3.7% vs. 4.9%; HR 0.77 (95% CI 0.64, 0.93), *p* = 0.0070) and all-cause mortality (4.3% vs. 5.8%; HR 0.76 (95% CI 0.64, 0.90), *p* = 0.0020) [3,13].

As mentioned, randomized trials have shown the superiority of prasugrel and ticagrelor over clopidogrel in patients with acute coronary syndromes, and both drugs are considered a class I recommendation for use in patients who have ACS.

However, the design of these studies was imperfect: ticagrelor was given as a pretreatment drug before PCI, while prasugrel was given after knowledge of coronary anatomy, so the design compared different strategies rather than randomizing different drugs for the same indication [5]. Moreover, a study conducted in Japan, South Korea, and Tiwan showed more bleeding in treatment with ticagrelor vs. clopidogrel, but could not reach statistical significance regarding efficacy between the two groups [14]. Similarly, Gimbel et al. found that ticagrelor was associated with a higher bleeding rate but had similar efficacy compared to clopidogrel [15]. However, while some trials showed the superiority of prasugrel over ticagrelor, other trials and meta-analyses failed to reach the same conclusion [9,16,17,18,19,20,21,22,23,24].

This study aims to address and compare the efficacy and safety of prasugrel and ticagrelor in patients with ACS. We hypothesize that both drugs are equally effective in preventing recurrent cardiovascular events and related mortality and are equally safe regarding major bleeding.

## 2. Methods

### 2.1. Study Population

A retrospective, observational, single-center study included patients 18 years or older who were admitted to the Cardiology Department at Bnai Zion Medical Center, Haifa, Israel, between January 2010 and December 2019 with acute coronary syndrome (ACS).

Patients were identified based on the primary diagnosis as classified by the International Classification of Diseases, Tenth Revision (ICD-10):

- Unstable angina pectoris—ICD-10 code I20;
- ST elevation myocardial infarction (STEMI)—ICD-10 codes I21.0/I21.1/I21.2 or I21.3;
- Non-ST elevation myocardial infarction (NSTEMI)—ICD-10 code I21.4.

Then, each case was reviewed manually by two different physicians to identify inclusion and exclusion criteria. In case of disagreement between the physicians, a third physician reviewed the case.

Medical management and treatment: antiplatelet medication for each patient was tailored personally in a case-by-case manner by the attending physician, based on up-to-date guidelines, available data, risk-benefit consideration, and their expert opinion.

### 2.2. Inclusion Criteria

All patients, 18 years or older, were hospitalized with the primary diagnosis of acute coronary syndrome, underwent percutaneous coronary intervention (PCI) during index hospitalization, and were treated with either prasugrel or ticagrelor. All consecutive patients who met the inclusion criteria and none of the exclusion criteria during the study period were included in the study.

### 2.3. Exclusion Criteria

Patients with a primary diagnosis other than acute coronary syndrome (ACS), those diagnosed with secondary myocardial infarction (Type II MI), those who eventually did not undergo PCI during index hospitalization, those who were not treated with ticagrelor or prasugrel, pregnant women, and those younger than 18 years were excluded.

### 2.4. Study Endpoints

The primary endpoint was defined a composite of cardiovascular death, recurrent ACS, or ischemic stroke during the one year of treatment for DAPT (3P-MACE: 3-point major adverse cardiovascular events).

The secondary endpoints were defined as the individual endpoints above (cardiovascular death, recurrent ACS, or ischemic stroke in one year) or major bleeding according to the BARC classification (Type 3, 4, and 5).

### 2.5. Data Collection

All data were collected from the MAX^®^ software patient management system that was used in Bnai Zion Medical Center during the period of the study. Clinical and lab data before and after index hospitalization were collected from the OFEX^®^ system for community patient management and were completed by phone surveillance where needed.

Mortality data were continuously updated by the Ministry of Interior; thus, all mortality data were available regarding all patients.

The following parameters were noted: demographic data and concomitant diseases including chronic obstructive disease (COPD), hypertension, diabetes mellitus, ischemic heart disease, chronic kidney disease, peripheral arterial disease, atrial fibrillation, valvular heart disease, and pulmonary hypertension. In addition, regular relevant medical therapy, laboratory results including hemoglobin, creatinine, and electrolytes on admission, and recommended treatments on discharge were also collected.

The study was approved by the local Institutional Review Board (IRB protocol approval number “0074-20-BNZ”). Patients’ informed consent was waived by the IRB Committee due to the nature of the study.

### 2.6. Statistical Analysis

SPSS^®^ software version 26 was used for statistical analysis.

Continuous variables were visually inspected for a normal distribution and are summarized as mean ± standard deviation. Categorical variables are presented with frequencies and proportions.

For the comparison of continuous quantitative variables (age, creatinine values, and hemoglobin) a non-paired, two-tailed student *t*-test was used. A *p*-value < 0.05 was considered a statistically significant value.

To examine the relationship between two categorical variables such as demographic characteristics and diagnosis, a chi-square or Fisher test was used.

In all statistical tests, a difference with a significant level was defined as a value of *p* < 0.05. The calculated sample size to detect between-group difference with 80% power, 0.05 significance level, and binary outcome non-inferiority, based on the previous literature incidence rate [5], was 704 patients. All data analyses, including the analysis of the primary endpoint, were performed according to the intention-to-treat principle.

## 3. Results

**Figure 1:**
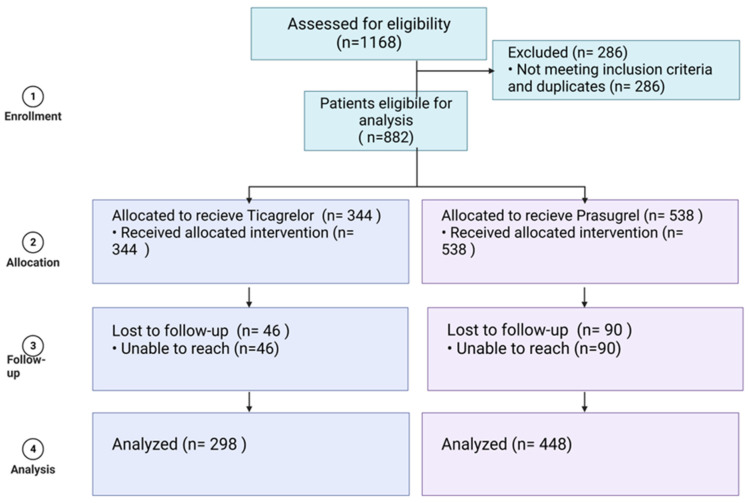
Patient inclusion and exclusion flow chart.

Between January 2010 and December 2019, 1168 patients were admitted with acute coronary syndrome at Bnai Zion Medical Center Cardiology Department, Haifa, Israel, and discharged with a recommendation for treatment with either prasugrel or ticagrelor in addition to aspirin. Among them, a total of 286 patients did not meet the inclusion criteria or met one or more of the exclusion criteria: 32 patients died during index hospitalization after PCI (these patients were treated with aspirin and either prasugrel or ticagrelor until they died during the hospitalization), 118 did not undergo PCI during their hospitalization, and 136 had secondary myocardial ischemia. Out of the eligible 822 patients, full data were available for 746 patients, who were included in the final analysis (Figure 1).

Patients were divided into two groups, prasugrel (448 patients) vs. ticagrelor (298 patients) treatment (*p* < 0.001). All the patients received aspirin as well. In both groups, most patients were male: 89.1% in the prasugrel group vs. 84.9% in the ticagrelor group (*p*-value = 0.001)

The prasugrel group patients were younger compared to the ticagrelor group patients, with average ages of 57.4 and 61.5, respectively (*p*-value < 0.001).

However, cardiovascular risk factors were comparable between the two groups, including diabetes, insulin use, smoking status, arterial hypertension, hypercholesterolemia, and familial history of coronary artery disease (CAD). Similarly, cardiac disease history including previous myocardial infarction and cardiac or aortic surgery were similar in both groups. Moreover, other relevant pre-inclusion characteristics and tests, such as weight, height, blood pressure on admission, and hemoglobin and creatinine tests on admission were similar in both groups (Table 1).

A significant difference in the incidence of diagnosis between the two groups was noticed: STEMI was the leading diagnosis in 393 (87.7%) patients in the prasugrel group, compared to 47 (15.7%) patients in the ticagrelor group (*p*-value < 0.001). Non-STEMI was the main diagnosis in 203 (68.1%) patients in the ticagrelor group versus 28 (6.3%) patients in the prasugrel group (*p*-value < 0.001). Unstable angina pectoris was the primary diagnosis in 27 (6%) and 48 (16.1%) patients in the prasugrel group and ticagrelor group, respectively.

## 4. Primary Endpoint

The incidence of the primary endpoint (a composite of cardiovascular death, recurrent ACS, or ischemic stroke during the one year of treatment with DAPT) was reached in a total of 70 patients (9.4%) in our cohort (Table 2). The event rate was similar in both groups: 8% and 10.3% in the ticagrelor group and prasugrel group, respectively. The relative risk was 1.01 (95% CI: 0.61, 1.64); *p* = 0.303.

Similarly, the secondary efficacy outcomes were achieved at similar rates in both the prasugrel and ticagrelor groups.

There were no cases of death due to cardiovascular causes in the ticagrelor group, compared to five cases in the prasugrel group, with no statistical significance in the relative risk, at 0.14 (95% CI 0.01,2.45); *p* = 0.13. The rate of recurrent MI was 7.7% in the ticagrelor group and 7.8% in the prasugrel group, with a relative risk of 0.98 (95% CI 0.6,1.6); *p* = 0.9. The incidence of stroke was 0.3% in the ticagrelor group and 1.3% in the prasugrel group, with a relative risk of 0.25 (95% CI 0.03,2.07); *p* = 0.06.

In addition, a subanalysis of primary endpoints was performed according to various admission diagnoses: there was no effect of primary diagnosis on the primary endpoints in both groups of the study, with *p*-values of 0.995, 0.621, and 0.440 for the STEMI, non-STEMI and UAP groups, respectively (Table 3).

Furthermore, the safety endpoint was achieved at similar rates in both ticagrelor and prasugrel groups. The occurrence of major bleeding events (BARC 3-5) was seen in nine patients (3.01%) in the ticagrelor group and in thirteen (2.9%) patients in the prasugrel group. The difference was not statistically significant (*p* = 0.932) (Table 4).

Discontinuation of new antiplatelet therapy before completion of the year of treatment after coronary intervention was observed more in ticagrelor-treated patients (n = 50, 16.72%) than in prasugrel-treated patients (n = 43, 9.59%) and *p* = 0.003.

The leading reasons for discontinuation of treatment in the prasugrel group were bleeding and a lack of adherence (28% and 21%, respectively), while in the ticagrelor group, they were adverse effects and bleeding (26% and 24%, respectively) (Table 5).

## 5. Discussion

In all ACS patients, a P2Y12 receptor inhibitor is recommended in addition to aspirin, given as an initial oral loading dose followed by a maintenance dose for 12 months, unless there is a high bleeding risk or unless the patient needs urgent surgery or oral anticoagulant therapy. Therefore, dual oral antiplatelet therapy for acute coronary syndrome with more potent antiplatelet agents (ticagrelor and prasugrel) is a cornerstone therapy for reducing recurrent atherothrombotic and major cardiovascular events [9,25].

**Figure 2:**
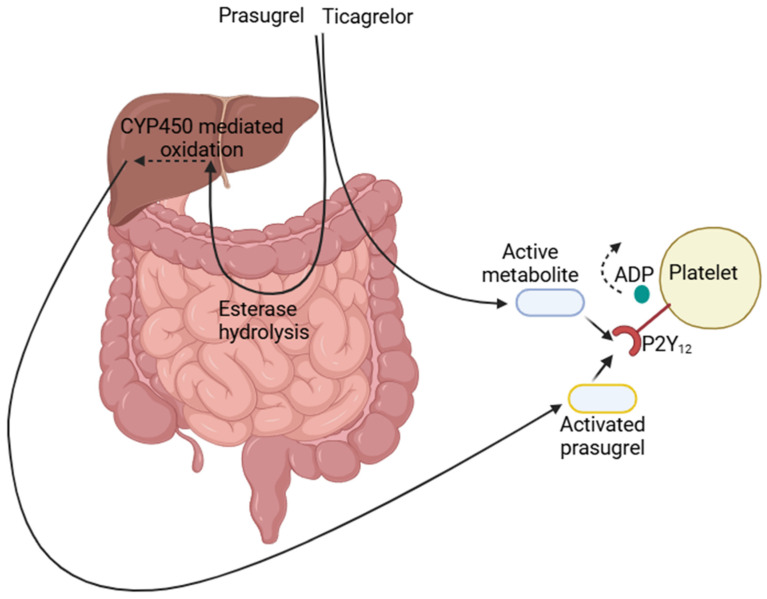
Pathways of prasugrel and ticagrelor to platelet inhibition.

The introduction of prasugrel and ticagrelor was a landmark in antiplatelet therapy that has redefined pharmacotherapy for ACS. Both drugs are pivotal in ACS therapy, having enhanced the efficacy of antiplatelet strategies. However, their action methods are quite different and have paramount consequences for clinical practice: prasugrel is a prodrug and requires enzymatic metabolism, mainly by carboxylesterases and cytochrome P450, for the generation of its active form, R-138727. This metabolite irreversibly binds to P2Y12-A-DP receptors on platelets and effectively inhibits ADP-mediated activation of platelets. In contrast, ticagrelor is not a prodrug, it directly and reversibly binds to the P2Y12 receptor, blocking ADP binding and preventing platelet activation without permanently altering the receptor. This reversible interaction enables quicker recovery of platelet function following discontinuation of treatment compared to prasugrel [11,13] (Figure 2). Although the time to the plasma concentration peak (Cmax) after the prasugrel dose was 30 min [26,27], while the time to Cmax with ticagrelor was 3 h [28], a direct comparison randomized study concluded that ticagrelor produced significantly higher platelet inhibition compared to prasugrel. At the end of the study timepoint (day 15), platelet reactivity was lower for ticagrelor [24].

Despite the pharmacologic differences, activation and mechanism of action, our data shows that both prasugrel and ticagrelor demonstrate similar clinical efficacy in reducing ischemic events in the context of ACS, despite differences in the registration trials mentioned earlier. Furthermore, clinical trials, including TRITON-TIMI 38, have shown that prasugrel significantly decreases rates of death from cardiovascular causes, nonfatal myocardial infarction, and nonfatal stroke compared to clopidogrel, although at the expense of a higher risk of major bleeding [11]. Similarly, ticagrelor has been associated with reduced rates of adverse cardiovascular events, demonstrating effectiveness without the prolonged inhibition observed with prasugrel. Nonetheless, the increased bleeding risk remains a critical consideration for clinicians when choosing antiplatelet therapy, particularly in vulnerable populations such as the elderly or those with a history of cerebrovascular events. In other words, both prasugrel and ticagrelor serve essential roles in the management of ACS, with their differing mechanisms of action serving to tailor therapy according to patient-specific variables and risk profiles. However, in specific populations such as Asians or those older than 70, clopidogrel may be associated with lower bleeding rates than ticagrelor [14,15].

The Intracoronary Stenting and Antithrombotic Regimen: Rapid Early Action for Coronary Treatment (ISAR-REACT) 5 trial was the first trial to compared the efficacy and safety of two treatment strategies head-to-head in patients with ACS [9]. This study included 4018 ACS patients for whom an invasive evaluation was planned. A prasugrel-based strategy was superior to a ticagrelor-based strategy in reducing the incidence of death, myocardial infarction, or stroke at 1 year (6.9% vs. 9.3%, *p* = 0.006), with a number needed to treat of 42, without any increase in bleeding complications (4.8 vs. 5.4%, *p* = 0.46).

This result was driven by a significant reduction of 1.8% in the incidence of recurrent myocardial infarction, with no significant between-group difference in the incidence of major bleeding.

Nevertheless, the ISAR-REACT 5 trial did not compare two antiplatelet drugs for the same indication; rather, it compared two antiplatelet treatment strategies involving two different drugs: ticagrelor was given as a pretreatment drug before PCI, while prasugrel was given after knowledge of coronary anatomy [9,11,25,29]. In addition, there were some important limitations of the ISAR-REACT 5 study, including the lack of a double-dummy design, approximately 19% of the patients not receiving a trial drug at discharge, and the fact that events were predominantly ascertained through telephone contact with the patients [9]. However, a subgroup analysis of the ISAR-REACT5 concluded that both drugs are equally effective in patients with diabetes mellitus [30]. Following the ISAR-REACT, some trials concluded that prasugrel is superior to ticagrelor, with these trials being designed similarly to the ISAR-REACT [16,17,31]; however, other trials showed no difference between the two medications [18,19,32]. Indeed, a meta-analysis including about 95,000 patients found no difference in outcomes among ACS patients treated with either medication [20]; other meta-analyses showed similar conclusions [21,22], while one meta-analysis concluded that prasugrel might have a better efficacy profile than ticagrelor in patients with ACS undergoing PCI. However, this advantage was only seen in pooled observational studies and is likely to be affected by selection bias [23].

Influenced by the results of the ISAR-REACT 5, the 2020 ESC Guidelines for the management of acute coronary syndromes in patients presenting without persistent ST-segment elevation recommend that prasugrel be considered in preference to ticagrelor for NSTE-ACS patients who proceed to PCI [25].

Furthermore, long-term data and additional comparative effectiveness data between the two potent P2Y12 drugs are lacking.

Our study compared prasugrel and ticagrelor head-to-head as drugs for the same indication, not as different strategy drugs. In comparison to the ISAR- REACT 5 trial, which showed a treatment benefit with prasugrel over ticagrelor, in this study, there was no statistically significant difference in the occurrence of recurrent cardiac events, stroke, or cardiovascular death between the two groups. Similar results were seen in the SWEDEHEART study, which was among the leading large-scale studies to compare prasugrel and ticagrelor in a retrospective trial and showed no difference in the one-year safety and efficacy of these drugs in patients with myocardial infarction treated with PCI [33].

In terms of the occurrence of significant bleeding, there was also no demonstrated statistically significant difference between the two groups. Both new antiplatelet drugs have been shown to be effective and safe, with an occurrence rate of events similar to large studies in the field.

It was found that at 1-year follow-up, more patients who were receiving ticagrelor at discharge had discontinued the trial therapy than those treated with prasugrel. The reasons for discontinuation of the drug in both groups were allergies, non-adherence to the drug therapy due to low compliance, the need for oral anticoagulation, bleeding, and plans to undergo CABG. This relatively high rate is due to more patients experiencing dyspnea and more noncompliant patients in the ticagrelor group compared to prasugrel group.

Regarding the lack of adherence to therapy, our study shows a similar pattern, although different rates in comparison to the ISAR-REACT 5 study: our study shows a discontinuation rate for prasugrel and ticagrelor of 9.6% and 16.8%, respectively, while the rates were 12.5% vs. 15.2%, respectively, in the ISAR-REACT 5. In the SWEDEHEART trail, prasugrel and ticagrelor discontinuation rates were similar, at about 20% in each group.

It should be emphasized that in our institution, our common practice is to prescribe prasugrel to STEMI patients, ticagrelor to NSTEMI patients, and clopidogrel to UAP patients or to those who are contraindicated to prasugrel or ticagrelor. Prasugrel group patients are younger because this drug is not given to populations older than 80 years old according to the guidelines; however, the decision is made in a case-by-case manner, based on the attending physician’s clinical decision, as mentioned before.

In this study, there are some limitations:

1. This is a single-center retrospective study. Some patients were excluded because of incomplete data, and about 15% of the patients were lost to the one-year follow-up, which might have affected the data. Furthermore, there were more patients on prasugrel than ticagrelor, yet this is within the calculated sample size, powered to detect efficacy difference, and results from clinical judgment based on the current guidelines.
2. Because of the retrospective nature of the study, the study may have been affected by selection bias. Furthermore, some differences were seen between the two groups’ baseline characteristics. The patients in the prasugrel group were younger and the absolute majority of them were admitted with a diagnosis of myocardial infarction with ST-segment elevation, while in the ticagrelor group, a diagnosis of myocardial infarction without ST elevations was more common. This was implemented due to the acceptable department protocols at that time, which allowed treatment of younger patients (up to the age of 75) with a myocardial infarction and ST elevations with prasugrel, while older patients or those who were admitted due myocardial infarction without ST elevations were to be treated with ticagrelor. At the same time, there was a large group of patients with unstable angina who were not treated with potent antiplatelets but with clopidogrel. This group was not included in this study. So, it is not possible to rule out a certain bias that may exist in favor of each tested drug.
3. The management of patients who discontinued the drug before the end of the year was not evaluated. We do not have enough data about why and when the treatment was stopped.

Despite these limitations, the results of our study are consistent with well-known large randomized studies in the field such as the TRITOM TIMI 38, PLATO, and ISARREACT 5. The rate of the occurrence of 3P-MACE events and life-threatening bleeding is similar in these three studies and in our study. Also, it should be emphasized that in this study, a direct comparison was performed between prasugrel and ticagrelor.

Although the ISAR-REACT 5 study was a groundbreaker and changed many accepted concepts, a question arises regarding whether it is possible to make a drastic change in the therapeutic approach and guidelines based on one study with some major discrepancies in real-life practice. Our study is among the first real-life ones to directly compare safety and efficacy between prasugrel and ticagrelor, and with cumulative data from other real-world studies such as the SWEDEHEART [33] mentioned earlier, there is no doubt that additional randomized multi-center studies to optimally investigate the issue will follow, especially now that pre-treatment is not recommended in most cases.

In conclusion, among patients hospitalized due to an acute coronary event and treated for a year with potent antiplatelet drugs, there was no statistically significant difference in the composite occurrence of recurrent heart events, stroke, and cardiovascular death between the two groups, nor in terms of the occurrence of significant bleeding. Both new antiplatelet drugs were shown to be effective and safe, with an event rate similar to large studies in the field; however, the early discontinuation rate is higher for ticagrelor than prasugrel.

## Figures and Tables

**Table 1:** Baseline characteristic of the study population.

	Prasugrel Group (n = 448)	Ticagrelor Group (n = 298)	*p*-Value
Age (years)	57.4 ± 10.2	61.5 ± 10.3	*p* < 0.001
Female			
N (%)	49 (10.9)	45 (15.1)	*p* = 0.001
Cardiovascular risk factors–no. (%):			
Diabetes	130 (29)	91 (30)	0.67
Current smoker	221 (49.3)	146 (49)	0.89
Past smoker	63 (14)	41 (13.7)	0.89
Arterial hypertension	258 (57.6)	174 (58.3)	0.87
Hypercholesterolemia	292 (65.2)	197 (66.1)	0.84
Family history of CAD	99 (22)	63 (21.1)	0.73
Background Medications–no. (%)			
Insulin	41 (9.1)	27 (9)	0.98
ACE-I/ARBs	261 (58.2)	179 (60)	0.62
Beta Blockers	88 (29.5)	149 (33.2)	0.28
Calcium Channel Blockers	102 (34.2)	165 (36.8)	0.46
Aspirin	158 (53)	228 (50.1)	0.57
Medical history–no. (%):			
Myocardial infarction	97 (21.6)	68 (22.8)	0.72
Aortocoronary bypass surgery	11 (2.4)	6 (2)	0.68
Blood pressure–mmHg			
Systolic	132 ± 5.6	130.8 ± 8.2	0.55
Diastolic	77.6 ± 4.6	77.8 ± 4.1	0.999
Weight (kg)	85.5 ± 7.7	84 ± 6.2	0.999
Height (cm)	175 ± 10.3	173 ± 8.6	0.26
Hemoglobin level (g/dL) *	13.9 ± 1.5	13.9 ± 1.6	0.99
Creatinine level (mg/dL) **	0.9 ± 0.4	0.9 ± 0.3	0.99
Diagnosis at admission–no. (%):			*p* < 0.001
STEMI	393 (87.7)	47 (15.7)	
NON-STEMI	28 (6.3)	203 (68.1)	
UAP	27 (6)	48 (16.1)	

* Hemoglobin level was not available for 9 patients. ** Creatinine level was not available for 12 patients. STEMI = ST-segment elevation myocardial infarction. NON-STEMI = non-ST-segment elevation myocardial infarction. UAP = unstable angina pectoris. Plus and minus values are means ± standard deviation.

**Table 2:** Primary and secondary endpoints.

	Drug				
	Ticagrelor (n = 298)	Prasugrel (n = 448)	Total (n = 746)	*p*-Value	Relative Risk (95% CI)
Primary outcome					
(Composite of death from a CV cause, MI, or stroke)–no. (%)	24 (8.0)	46 (10.3)	70 (9.4)	0.303	1.01 (0.61, 1.64)
Secondary outcomes					
CV death–no. (%)	0 (0)	5 (1.1)	5 (0.7)	0.13	0.14 (0.01, 2.45)
MI–no. (%)	23 (7.7)	35 (7.8)	58 (7.7)	0.9	0.98 (0.6, 1.6)
Stroke–no. (%)	1 (0.3)	6 (1.3)	7 (0.9)	0.06	0.25 (0.03, 2.07)
Major bleeding					

MI = myocardial infarction; CV = cardiovascular.

**Table 3:** Primary endpoint comparison based on diagnosis.

	Primary Endpoint Ticagrelor Group (n = 24)	Primary Endpoint Prasugrel Group (n = 46)	*p*-Value
STEMI No. (%)	5 (20.8)	40 (87)	0.995
NSTEMI No. (%)	16 (66.7)	3 (6.5)	0.621
UAP No. (%)	3 (12.5)	3 (6.5)	0.44

STEMI: ST elevation myocardial infarction; NSTEMI: non-ST elevation myocardial infarction; UAP: unstable angina pectoris.

**Table 4:** Secondary endpoint.

	Drug			
	Prasugrel (n = 448)	Ticagrelor (n = 298)	Total (n = 746)	*p*-Value *
**Secondary endpoint η–** **no. (%)**	13 (2.9)	9 (3)	22 (2.9)	0.9 NS ‡

η—defined as incidence of major bleeding (BARC type 3 through 5); ‡—non-significant; *—according to chi-square test.

**Table 5:** Treatment discontinuation data including etiology.

	Prasugrel	Ticagrelor	Total
**Allergy**	4	2	6
**Adverse effects**	2	13	15
**Underwent CABG**	3	5	8
**Indication for oral anticoagulation**	1	5	6
**Lack of adherence/medication stopped for medical reason**	9	13	22
**CVA/TIA**	4	0	4
**Bleeding**	12	12	24
**Died**	8	0	8
**Total**	43 (9.6%)	50 (16.8%)	93

CABG: coronary artery bypass grafting; CVA: cerebrovascular attack; TIA: transient ischemic attack.

## Data Availability

The coded data is available upon direct request to the corresponding authors.
